# Bilateral Renal Cortical Necrosis in Meningococcal Meningitis

**DOI:** 10.1155/2011/274341

**Published:** 2011-11-22

**Authors:** C. Kennedy, S. Khilji, A. Dorman, J. Walshe

**Affiliations:** ^1^Department of Nephrology, Beaumont Hospital, Dublin 9, Ireland; ^2^Department of Pathology, Beaumont Hospital, Dublin 9, Ireland

## Abstract

Bacterial meningitis is a relatively common infection of the cerebrospinal fluid (CSF) and leptomeninges. The clinical picture evolves rapidly and, if treatment is delayed, can result in a variety of long-term sequelae, including death. Acute kidney injury in the setting of bacterial meningitis usually results from hypotension and volume depletion and resolves with appropriate treatment. Meningococcaemia with profound hypotension, and/or disseminated intravascular coagulopathy (DIC) may very rarely lead to bilateral renal cortical necrosis. In this context, renal recovery is extremely unlikely. We present two cases of meningococcaemia complicated by bilateral renal cortical necrosis and, ultimately, end stage kidney disease. We also present a review of the literature on the subject. The cases outline the importance of early aggressive intervention by a multidisciplinary team.

## 1. Introduction

Bacterial meningitis is a bacterial infection of the CSF associated with inflammation of the leptomeninges. It is a relatively common infection with over 1.2 million cases annually worldwide [1]. The classic presenting features are fever, nuchal rigidity, and altered mental status. Rapid symptom evolution is usually seen, and patients may deteriorate neurologically and systemically in a matter of hours.

Diagnosis relies on CSF sampling by means of lumbar puncture [2]. CSF analysis in this setting typically reveals a neutrophil leucocytosis with an elevated protein concentration and a reduced glucose concentration. The most common causative organisms are *Neisseria meningitidis *(subgroups A, B, C, W135, Y, and Z)*, Streptococcus pneumonia, *and* Haemophilus influenzae.*

Since the advent of antibiotics, bacterial meningitis is no longer universally fatal. However, an appreciable mortality and morbidity remains, particularly with pneumococcal infections [3]. Neurological complications include sensorineural hearing loss, focal neurological deficits, and intellectual impairment [4]. Nonneurological complications are usually seen in the short term and include adult respiratory distress syndrome, reactive arthritis, acute kidney injury, disseminated intravascular coagulation (DIC), and Waterhouse-Friedrichsen Syndrome.

Severe bacterial meningitis may rarely lead to a variety of renal complications including acute kidney injury, oliguria, and rhabdomyolysis [5]. Persistent renal failure should lead to the suspicion of bilateral renal cortical necrosis. Fluid resuscitation and, if indicated, timely inotropic support remain the mainstay of prevention and treatment of these complications. Plasmapheresis, although variably successful in several case reports [6], has not been subjected to rigorous clinical trials and cannot be recommended as first line therapy in this disease.

We describe two adult cases of meningococcaemia resulting in irreversible kidney failure due to bilateral renal cortical necrosis. We also review the literature on the subject.

## 2. Case 1

A 21-year-old female presented to the emergency department with a 24-hour history of disorientation and a reduced level of consciousness. She had no significant past medical history. On examination, she was pyrexial and profoundly hypotensive, with an extensive petechial rash. Her Glasgow Coma Scale was 8/15, with nuchal rigidity and photophobia. Her laboratory investigations are shown in Table 1. She was transferred to the intensive care unit and managed with empiric antibiotics, fluids and inotropes for presumed meningococcal septicaemia.

Within hours, her blood cultures confirmed meningococcaemia with group B *Neisseria meningitidis*; later subtyped as NT : P1.9 : NT. She had not previously received the meningitis vaccine.

Despite large volume fluid resuscitation, the patient remained anuric. Within hours, she required continuous renal replacement therapy for volume control and management of her worsening acidosis.

**Figure 1:**
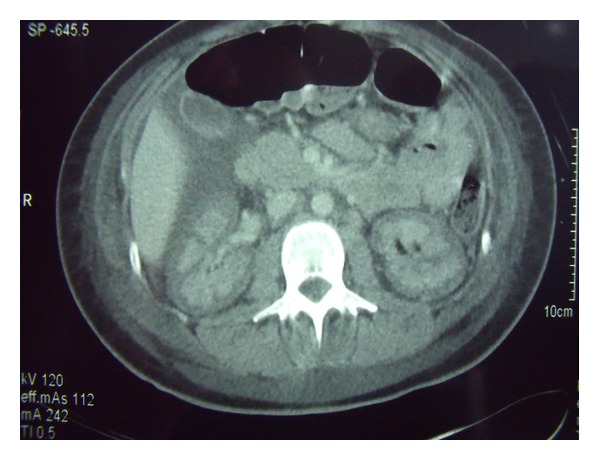
Computed tomography (CT) scan showing bilateral renal infarcts.

Over a period of days she slowly improved allowing extubation and weaning of her inotropic support. She remained anuric and dependent on intermittent haemodialysis. However, a contrast-enhanced abdominal CT was compatible with bilateral renal infarcts (Figure 1) without adrenal haemorrhage.

A cerebral CT scan revealed high signal adjacent to the choroid plexus consistent with a small intraparenchymal haemorrhage. Despite this, an excellent neurological recovery was made and the patient was discharged home two weeks after her initial presentation.

An elective renal biopsy one-month later revealed acute ischaemic infarction involving 50% of the sampled cortex with adjacent secondary acute tubular necrosis.

One year later, the patient reported increased urine volumes. A 24-hour urine collection, amounting to 1.6 litres of urine, quantified her creatinine clearance at 22 mL/min and dialysis was successfully tapered. Within six months, however, renal replacement therapy was once again necessary and, after three further months on dialysis, she was successfully transplanted.

## 3. Case 2

A 19-year-old male with no past medical history was transferred from an outside hospital with profound meningococcal septicaemia and secondary multiorgan failure, but without evidence of DIC (Table 1). He had presented hours previously with headache, rash, and confusion and rapidly deteriorated becoming profoundly hypotensive.

His blood and CSF cultures yielded group B *Neisseria meningitidis*. He had not been vaccinated against meningitis.

On arrival, he was intubated and heavily inotrope dependent. He was anuric and required continuous renal replacement therapy. He remained in this critical condition for two weeks before achieving sufficient haemodynamic stability to tolerate intermittent dialysis.

A slow recovery ensued. A significant period of inpatient rehabilitation was necessary. At the time of discharge, the patient was dialysis.

**Figure 2:**
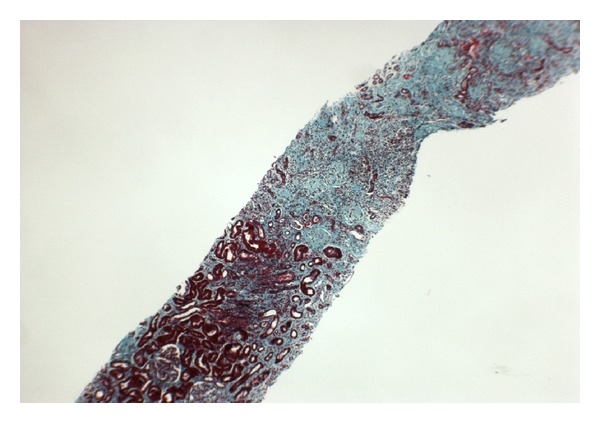
Renal biopsy viewed at low power stained with Trichrome Stain. There is severe fibrosis with subcapsular and cortical necrosis.

An elective renal biopsy was performed three months after initial presentation. This showed significant fibrosis with seventeen of twenty glomeruli sclerosed, with subcapsular and cortical necrosis (Figure 2). From a histological perspective, recovery was deemed highly unlikely. Transplant workup was initiated. A successful living donation was completed several months later.

## 4. Discussion

Bilateral renal cortical necrosis is a rare phenomenon. It follows a major vascular insult to the small arteries supplying the renal cortex, such as profound hypotension or a systemic coagulopathy. Endothelial injury, either in severe renal ischaemia or primary DIC, is seen as the initiating event. Interestingly, the patient in Case 1 had clinical and biochemical evidence of DIC; the patient in Case 2 did not.

Over half of female cases of cortical necrosis are pregnancy related, in situations such as placental abruption, severe preeclampsia or amniotic fluid [7]. Other causes include sepsis, drugs, trauma, and pancreatitis [8, 9].

Contrast-enhanced computed tomography (CT) is the imaging modality of choice and demonstrates hypodense areas in the renal cortex [10, 11]. Abdominal radiograph may show thin cortical shells caused by calcification. This, however, does not develop for many months.

Renal biopsy enables a definitive diagnosis and provides important prognostic information. There are five categories, based on the severity of the histopathological findings. In order of increasing severity, there are focal, minor, patchy, gross, and confluent forms of the condition.

No specific therapy has been shown to be effective in acute cortical necrosis. Many patients require renal replacement therapy. A minority (20 to 40%) partially recover with a creatinine clearance that stabilizes between 15 and 50 mL/min [12].

Acute kidney injury in the setting of bacterial meningitis incurs a high mortality rate [5]. Survival with bilateral acute cortical necrosis in this setting is rarely observed [13–15]. However, the two cases outlined above demonstrate that, despite the development of bilateral renal cortical necrosis, long-term patient survival is achievable with early aggressive intervention.

Meningitis, especially meningococcal meningitis, must always be treated as a medical emergency. Early aggressive management with fluids, antibiotics, and vasoactive agents is crucial in preventing prolonged hypotension and DIC which can ultimately lead to bilateral cortical necrosis. Although partial recovery may be experienced in bilateral renal cortical necrosis, as seen in Case 1, end-stage kidney disease is the likely outcome.

## Figures and Tables

**Table 1:** Admission laboratory investigations.

	Case 1	Case 2
Urea (mmol/L)	14.2	16.3
Creatinine (umol/L)	192	212
White cell count (×10^9^/L)	15	17.3
Hemoglobin (g/dL)	11.7	12.1
Platelets (×10^9^/L)	525	325
APTT (sec)	44	26
Fibrinogen (g/L)	0.52	2.73
